# Analysis of Chaperone mRNA Expression in the Adult Mouse Brain by Meta Analysis of the Allen Brain Atlas

**DOI:** 10.1371/journal.pone.0013675

**Published:** 2010-10-28

**Authors:** Andrew T. N. Tebbenkamp, David R. Borchelt

**Affiliations:** Department of Neuroscience, SantaFe Health Alzheimer's Disease Center, McKnight Brain Institute, University of Florida, Gainesville, Florida, United States of America; University of Cambridge, United Kingdom

## Abstract

The pathology of many neurodegenerative diseases is characterized by the accumulation of misfolded and aggregated proteins in various cell types and regional substructures throughout the central and peripheral nervous systems. The accumulation of these aggregated proteins signals dysfunction of cellular protein homeostatic mechanisms such as the ubiquitin/proteasome system, autophagy, and the chaperone network. Although there are several published studies in which transcriptional profiling has been used to examine gene expression in various tissues, including tissues of neurodegenerative disease models, there has not been a report that focuses exclusively on expression of the chaperone network. In the present study, we used the Allen Brain Atlas online database to analyze chaperone expression levels. This database utilizes a quantitative *in situ* hybridization approach and provides data on 270 chaperone genes within many substructures of the adult mouse brain. We determined that 256 of these chaperone genes are expressed at some level. Surprisingly, relatively few genes, only 30, showed significant variations in levels of mRNA across different substructures of the brain. The greatest degree of variability was exhibited by genes of the DnaJ co-chaperone, Tetratricopeptide repeat, and the HSPH families. Our analysis provides a valuable resource towards determining how variations in chaperone gene expression may modulate the vulnerability of specific neuronal populations of mammalian brain.

## Introduction

A common feature of the neurodegenerative diseases is the accumulation of misfolded and aggregated proteins in pathologic inclusion bodies in specific populations of neurons and, or, astroglia (for review see [1]). Recent studies in several model systems have suggested that accumulation of the pathologic protein aggregates imposes a burden on the protein homeostatic system [2], which includes the ubiqiutin/proteasome system, autophagic clearance systems, protein synthesis, and the chaperone network. Each of these systems functions in equilibrium to maintain the integrity of the proteome.

Among these systems, the protein chaperone network, which includes the heat shock proteins (HSPs), has received a significant amount of attention as a potential protective factor in neurodegenerative disease [3]–[5]. Chaperones can serve a variety of functions such as facilitating the folding of nascent proteins, refolding misfolded proteins, targeting proteins for degradation, preventing the aggregation of misfolded proteins, and transporting proteins across organelle membranes. Organized by molecular size and function, the families consist of, and are organized herein as, HSP70/HSPA, HSP40/DnaJ, HSP90/HSPC, Tetratricopeptide repeat domain (TPR) (or HSP 90 co-chaperones), small HSP (sHSP)/HSPB, HSP100 (which includes AAA+ ATPase proteins)/HSPH, HSP60 (chaperonins)/HSPD and HSPE, and Heat shock factors (HSF). Nomenclature used throughout this article follows that of the Cell Stress Society International, whenever possible [6]. Constituents of each family function via multiple mechanisms to regulate protein folding or mitigate the accumulation of misfolded proteins. The HSPAs and DnaJs work cooperatively in protein folding, utilizing ATP in the binding and release of substrates, with DnaJs stabilizing interactions of HSPAs with client proteins [7]. Similarly, HSPCs and their co-chaperones, identified by the presence of a TPR domain, coordinate client binding and ATP hydrolysis. The HSPBs, including the crystallins, mitigate protein aggregation by binding to exposed hydrophobic domains in misfolded clients to maintain solubility and assist in re-folding [8], [9]. The HSPH family and AAA+ ATPase proteins, impart a wide range of functions including proteins capable of exhibiting chaperone activity similar to the chaperonin family [10]. Chaperonins, with the guidance of Prefoldins, fold nascent polypeptides in a hetero-oligomeric chaperonin complex, which can encapsulate proteins that range in size up to 100 kDa [11]. The HSFs are upstream regulators of inducible HSP expression (for review see [4]).

One of the most poorly understood aspects of neurodegenerative disease involves the basis of cell and tissue vulnerability. In most of the inherited forms of neurodegeneration, the protein harboring the disease-causing mutation is ubiquitously or widely expressed, and yet often the pathology disproportionately affects specific populations of neurons. In diseases that are primarily disorders of the brain, specific substructures of the brain are either more vulnerable to accumulate misfolded proteotoxins, or in settings of widespread proteinopathy, specific substructures are more prone to degeneration. For example, in Alzheimer's disease inclusion bodies formed by Tau protein appear first in the entorhinal cortex before the pathology appears throughout the brain [12]. By contrast, Huntington's disease is characterized by severe degeneration of the caudate/putamen and cortex, with minimal degeneration of the cerebellum, despite near uniform expression of the disease protein, huntingtin, throughout the brain and widespread distribution of nuclear inclusion pathology [13], [14]. One potential mechanism to explain cell and tissue vulnerability could be related to diversity in the expression of protein chaperones across different neuronal populations and different tissues.

The transcriptional regulation of the HSPs is complex with examples of both constitutive and inducible regulatory mechanisms (for review see [15]). Previous studies have performed gene expression analysis of normal and diseased mouse CNS tissue which provide some clues to regional chaperone expression [16]–[25]. However, cross-experimental conclusions regarding expression levels are difficult to draw due to different sample preparations, controls, etc. Moreover, most of these studies have focused on changes in gene expression due to a disease-related insult. In the present study, we focus on characterization of the pattern of chaperone expression in specific substructures of the normal adult murine brain. Our aim was to establish a baseline expression of the chaperone network to provide a framework to explore the basis for neural vulnerability. To do this, we mined the on-line database of the Allen Brain Atlas (www.brain-map.org; [26]) as the source for all expression data. We then compiled the data on HSPAs, HSPCs, DnaJs and TPR domain proteins (co-chaperones to HSPAs and HSPCs, respectively), HSPBs, HSPHs, chaperonins, and HSFs and compared expression levels, as measured by *in situ* hybridization, across multiple structures of the brain. Our analysis reveals relatively little diversity in the expression patterns of chaperones, chaperonins, and the HSFs. By contrast, although less than one might have expected, the expression patterns of DnaJs and TPR domain proteins and the AAA+ ATPases showed greater diversity. Overall, this study provides a framework to investigate the extent to which diversity in the expression patterns of the heat shock proteins plays a role in the selective vulnerability of neuronal populations to specific misfolded proteins.

## Methods

### Database

The goal of the Allen Brain Atlas is to provide a “genome-wide map of gene expression in the mouse brain”. To accomplish this goal, coronal or sagittal tissue sections (fresh-frozen and unfixed, 25 µm thick) were probed by *in situ* hybridization (ISH) using sections spaced at intervals of 200 µm throughout the brain of 8-week old male C57BL/6J mice. Gene expression was visualized by ISH, using anti-sense probes generated with digoxigenin-labeled nucleotides. Bound probes were later visualized by adding the alkaline phosphatase substrate 5-bromo-4-chloro-3-indolyl phosphate (BCIP) then nitroblue tetrazolium (NBT). An in depth description of the high-throughput procedures and data acquisition can be found on the website (www.brain-map.org). The expression of over 20,000 genes, as of 2006, has been characterized. Unique gene entries were compiled first from the RefSeq database, followed by unique entries from TIGR and Celera databases and from the Riken FANTOM3 database. For each gene with a successful probe, raw images are accompanied by data showing quantified expression levels and densities. From this data we generated tables that provide a summary of HSP expression data.

### Data Analysis

To generate our database of heat shock proteins, we searched the NCBI database Clusters of euKaryotic Orthologous Groups (KOG) for HSPs and related homologues that are found in the human genome. Human protein accession numbers were used in the Basic Local Alignment Search Tool (BLAST) to find corresponding mouse HSPs. This list was cross-referenced with the Allen Brain Atlas database to ensure all HSPs were accounted for, including any that may not have been found from the initial KOG search. In other cases, namely the TPR domain proteins, the Picard lab website (http://www.picard.ch/downloads/downloads.htm), primary literature, and the NCBI Entrez Gene database was searched for empirical data for interactions or classifying motifs. The TPR family contains hundreds [27] of members, some of which may have no role in protein folding. Thus we focused on examining expression levels for the TPR family proteins with known function in protein folding.

Once we had assembled a gene list, we searched the Allen Brain Atlas database for expression data from the adult mouse brain. The data sets were organized by gene family as previously outlined. Of the 288 genes we identified in the mouse genome as constituents of the protein homeostasis network, expression data for 270 were found in the Allen Brain Atlas (Table 1 and Table S1). For some of the genes (e.g. Hsbp8) the database included 2 probe sets; in those cases both data sets were used in this meta-analysis. The ABA provides expression values from both coronal and sagittal sections, but although the values obtained from these different planes of section are similar in magnitude, they are not always identical. For consistency, we analyzed only data from sagittal sections. Abbreviations of the brain regions that were analyzed are: Forebrain: OFL, olfactory bulb; CTX, cerebral cortex; HIP, hippocampal region; HPF, hippocampal formation; RHP, retrohippocampal region; Basal Forebrain: LSX, lateral septal complex; STRv, striatum ventral region; STR, striatum; STRd, striatum dorsal region; PAL, pallidum; sAMY, striatum-like amygdalar nucleus; Midbrain: HY, hypothalamus; TH, thalamus; MB, midbrain; Hindbrain: P, pons; MY, medulla; CB, cerebellum.

**Table 1:** Summary of Total Chaperone Genes Analyzed.

Classification	HSPA	DnaJ	HSPC	TPR	HSPB	HSPH	Chaperonins	HSF	Other	Total
Total genes found	11	50	4	98	30	58	20	5	12	288
Number in ABA database	10	47	4	92	28	54	18	5	12	270
Expressed in brain	9	47	4	89	23	52	16	5	11	256
Regional Variation	5	30	1	52	13	30	8	2	4	145
Significant Variation	1	5	0	12	1	7	2	0	2	30

Chaperone genes are grouped into nine families. Each column shows the number of genes for each family, the number found in the ABA database, the number expressed in brain at some level, the number exhibiting any degree of variation across brain structures, and the number exhibiting significant variation (defined in text) across brain structures. Very few chaperones exhibit significantly varied expression levels.

### Western blot

Four C57BL/6J male mice at eight weeks of age were perfused with cold phosphate-buffered saline (PBS) followed by dissection on ice into eight brain regions: olfactory bulb (OLF), cortex (CTX), hippocampus (HIP), striatum (STR), thalamus and hypothalamus (TH/HY), midbrain (MB), pons and medulla (PN/MD), and cerebellum (CB). Brain tissue was homogenized by sonication in 10∶1 weight:volume Tris-buffered saline (20 mM Tris-HCl, 130 mM NaCl, pH 7.4). Homogenates were centrifuged at 3000× g for 5 minutes, and the supernatant was then mixed with sample buffer (60 mM Tris-HCl, 2% SDS, 0.01% bromophenol blue, 5% beta-mercaptoethanol). The mixed homogenates were sonicated again before boiling then loading onto 4–20% Tris-glycine polyacrylamide gels (Invitrogen, Carlsbad, CA) for SDS-PAGE. Following gel electrophoresis, proteins were transferred to nitrocellulose membranes, which were then incubated with antibodies against chaperones: DnaJb1, DnaJb2, and Tomm70a (Abcam, Cambridge, MA). The Gapdh antibody was a generous gift from Gerry Shaw (University of Florida). Western blots were imaged and bands quantified with a Fuji imaging system (FUJIFILM Life Science, Stamford, CT). Data was analyzed by a one-way ANOVA followed by a post-hoc Tukey test to determine statistical differences (GraphPad Prism 5.0, San Diego, CA).

## Results and Discussion

The expression data in the ABA database are assigned numerical values for strength of signal (relative to a positive control, dopamine receptor, Drd1a) with values ranging from 0 to 100. To make expression level comparisons between different regions of the brain, we grouped values in quintiles from highest to lowest expression and assigned each quintile a color code that is similar to the code used in heat maps: 81–100 (red), 61–80 (yellow), 41–60 (green), 21–40 (blue), >0–20 (gray), and 0 (white). Using the color codes, we generated a visually interactive representation of the expression levels of these genes across multiple brain regions.

Expression data for a total of 270 chaperone genes was identified in the Allen Brain Atlas database. The 270 genes examined include 14 chaperones (HSPA and HSPC), 139 co-chaperones (DnaJ and TPR domain), 94 accessory chaperones (HSPB, HSPH, and others), 18 chaperonins (including HSPD and HSPE), and 5 HSFs (Table 1). Nearly all of these genes, 256, were expressed at some level in brain; 13 chaperones, 136 co-chaperones, 86 accessory chaperones, 16 chaperonins, and 5 HSF genes. Of these 256 expressed genes, 145 show some level of variation, meaning that the level of expression ranks in different quintiles in at least one region of the brain (Table 1). It is important to note, however, that a difference of only one quintile ranking could be very small (possibly far less than 20 expression units). Thus, we considered a difference of only one quintile ranking as essentially equivalent. When we determined how many genes differ in expression level across different regions of the brain by ≥2 quintiles in ≥2 regions of the brain, only 30 (∼10%) of the genes qualify as showing variability in expression level across different regions of the brain (Table 1).

Notably, relatively few genes of the total chaperone network are ubiquitously expressed at high levels (Table 2, Table S2). By contrast, 95 genes showed ubiquitously low or no expression (Table 2, Table S2). Some of the 15 genes that are not expressed are known to be stress/heat inducible; however, it is sill noteworthy that approximately one-third of the genes in the chaperone network are expressed at relatively low levels in brain. Below, we summarize the findings of our analysis, first according to HSP class then by brain region and expression levels. Within each color-coded figure, brain regions are organized in a general rostral to caudal fashion. Regions were grouped into forebrain, basal forebrain, midbrain, and hindbrain. With regard to cell-type specificity, the ABA does not give the necessary resolution or markers indicating cell type for each probe. In general, we could expect that large cell bodies of neurons would be the source of most of the mRNA hybridization signal. Although the clients and targets of functionality for the vast majority of these chaperones in brain are unknown, we provide information regarding function whenever possible.

**Table 2:** Summary of Uniformly Expressed Genes.

Expression level	HSPA	DnaJ	HSPC	TPR	HSPB	HSPH	Chaperonins	HSF	Other	Total
Ubiquitously high	3	6	3	8	0	7	4	1	3	35
Ubiquitously low	1	11	0	33	11	14	4	2	4	80
Ubiquitously off*	1	0	0	3	5	2	2	1	1	15

This table shows the number of chaperone genes within each of the nine families that have ubiquitously high, low, or no expression across brain regions analyzed.

*Some genes with no expression are known to be heat/stress inducible. Approximately one-third of all chaperones exhibit low or no expression.

### HSPA (HSP70s)

**Figure 1:**
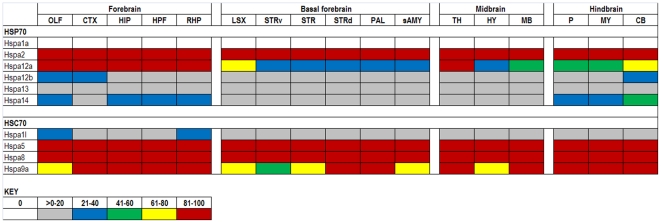
Expression analysis of HSPA family members. Gene expression levels of HSPA (HSP70) family members (rows) show little variability across 17 structures (columns, see text for abbreviations) of the adult mouse brain. Hspa1a is known to be an inducible heat shock gene.

The major chaperones to respond to cell stress are the HSPAs. Four of these are highly expressed throughout the brain (Figure 1). However, half of these ten genes are expressed at or below the 20^th^ percentile (1–20%). It is important to note that some of these poorly expressed genes include genes that are heat/stress inducible, namely Hspa1a the classic HSP70. Mice nullizygous for Hspa1a exhibit a deficit in protection against cell death after cerebral ischemia [28], TNF-α toxicity [29], or heart infarction [30] after heat or ischemia preconditioning, respectively. Only one of these HSPA genes, Hspa12a, shows significant variability in expression levels across the brain. Hspa12a does not have a well-defined function but has been shown to be decreased in cortical regions of schizophrenic patients [31], and increased in atherosclerotic lesions [32]. The classic Heat Shock Cognate 70 genes are all expressed at high levels with the exception Hspa1l, which is reported to be specifically expressed in spermatids [33]. HSC70s have functions consistent with classical chaperones including nascent polypeptide binding, but also function in fundamental cell processes such as uncoating of clathrin-coated vesicles [34] and in stabilizing nascent polypeptides targeted to the translocase machinery of the mitochondrial membrane (Timm44, see below) [35], [36]. Hspa8 (Hsc70) is considered to be the essential ‘housekeeping’ member of the 70 kD family which explains its high level of expression (reviewed in [37]).

### DnaJ (HSP40s)

**Figure 2:**
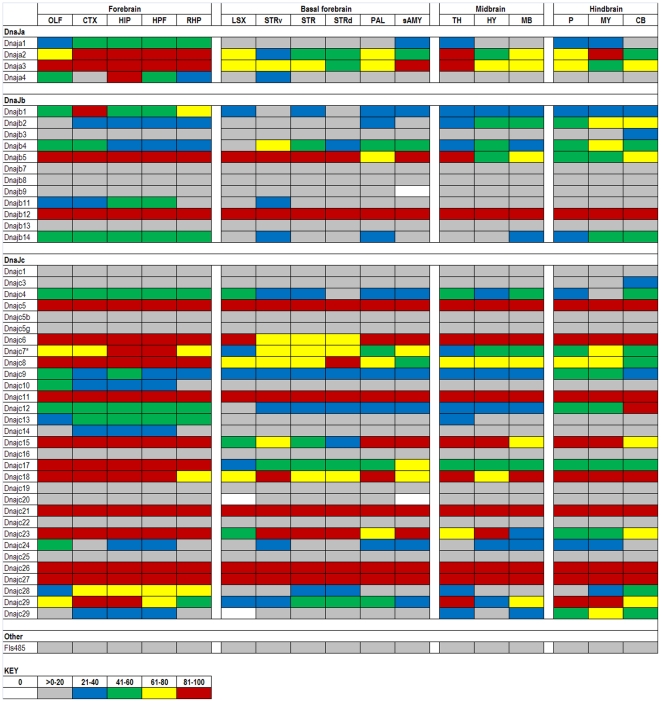
Expression analysis of DnaJ family members. Expression levels of DnaJ (HSP40) subfamilies A, B, C, and Others (rows) are shown across 17 structures (columns) of the adult mouse brain. More than half of the genes exhibit some variability across the brain, but few have significant differences (≥2 expression quintiles in ≥2 structures). DnaJc7 (*) is also known as Ttc2, a tetratricopeptide repeat domain co-chaperone. Duplicated genes are listed where two data sets were available from the ABA (DnaJc29).

The DnaJ genes encode members of the HSP40/DnaJ family, which largely function as co-chaperones for HSPAs. The expression pattern of these genes shows considerably more variability than the chaperone genes (Figure 2). Although all 47 of the DnaJ genes analyzed in the database show some level of expression, approximately one third express at levels below the 21^st^ percentile. The DnaJb subfamily members tend to show more consistent expression across brain regions, with several being consistently in the lower 20^th^ percentile. DnaJb1 and 2 show substantial variation in expression levels while the DnaJb12 gene, is consistently highly expressed across all brain regions. Similar to the DnaJb family, the DnaJc family contains many genes that exhibit consistently low levels of expression. The most substantial variation in expression is exhibited by DnaJc7, 28, and 29. The two probes of DnaJc29, however, do not produce the same pattern across each brain region (for reasons unknown), but both probes indicate variable expression. The DnaJc5, 11, 18, 21, 26, and 27 family members are consistently highly expressed. Overall, there are examples of both consistency and variability in DnaJ gene expression across brain regions.

In assessing the expression of all DnaJ genes within a particular brain region, we note that the DnaJa family of chaperones is rather highly expressed in forebrain, with lower levels in other brain structures (Figure 2). Genes within the DnaJb and DnaJc families as a whole are more consistently expressed between fore-, mid-, and hindbrain with a few individual genes showing a significant degree of variability.

### HSPC (HSP90s)

**Figure 3:**
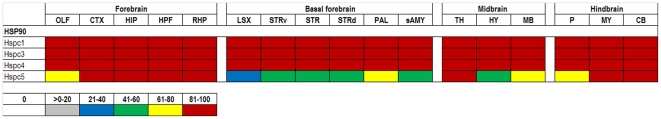
Expression analysis of HSPC family members. Gene expression levels of the HSPC (HSP90) chaperone family. All are near ubiquitously and highly expressed in the adult mouse brain.

Other chaperones that operate in the HSPA pathway are the HSPC family members. Three of the four HSPC family members are evenly and highly expressed throughout the brain at high levels (Figure 3). HSPCs function in two major regulatory processes: 1) the heat shock response via HSF [38] and 2) steroid hormone receptor stabilization (reviewed in [39]–[41]). In the first regulatory process, HSPC forms inhibitory complexes that sequester HSF and prevent its activation of chaperone transcription. After a cellular stress, HSPCs bind misfolded proteins thus releasing their inhibition of HSF and initiating a cellular response to the stress. In the second regulatory process, HSPC binds indiscriminately to unliganded steroid hormone receptors, stabilizing them for hormone binding, and once bound, modulates gene expression. Another HSPC member, Hspc5, exhibits a more variable expression pattern, but is generally highly expressed. Hspc5 is a mitochondrial chaperone that fails to bind standard HSPC co-chaperones Ptges3 and Hop, but is classified as an HSPC due to its high homology to Hspc1 and susceptibility to geldanamycin inhibition of ATP binding and hydrolysis [42]. Thus, the primary regulatory HSPC genes seem to be fairly uniformly expressed as might be expected. Knockout mice for Hspc4 die at 10 days of gestation from a failure of allantois to expand blood vessels once fused to the chorion [43].

### TPR

**Figure 4:**
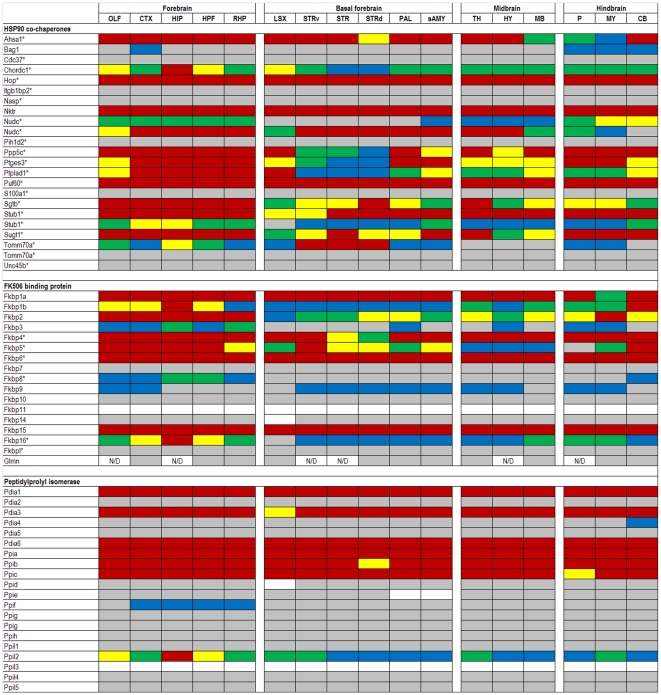
Expression analysis of TPR family members. Expression levels of tetratricopeptide repeat (TPR) chaperones. The TPR chaperones are divided into four subfamilies, HSPC co-chaperones (* represents proteins identified through Picard lab website; see text), FK506 binding protein (Fkbp), Peptidylprolyl isomerase (Ppi), and Tetratricopeptide repeat domain (rows). Approximately half of all TPR genes show some variability across 17 brain structures (columns). N/D indicates no data was available for those specific regions. Duplicated genes are listed where two data sets were available from the ABA.

**Figure 5:**
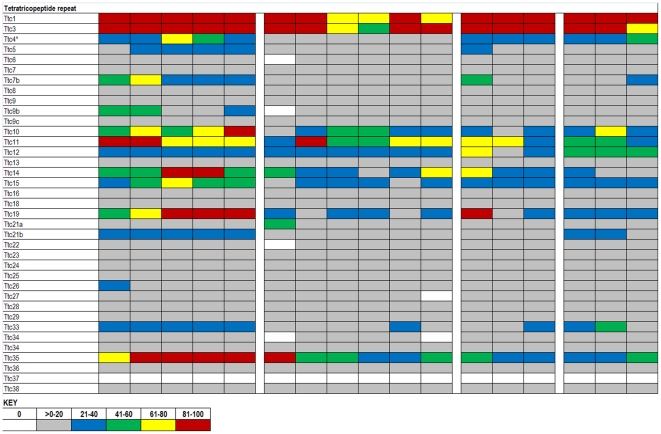
Expression analysis of TPR family members. Expression levels of tetratricopeptide repeat (TPR) chaperones. The TPR chaperones are divided into four subfamilies, HSPC co-chaperones (* represents proteins identified through Picard lab website; see text), FK506 binding protein (Fkbp), Peptidylprolyl isomerase (Ppi), and Tetratricopeptide repeat domain (rows). Approximately half of all TPR genes show some variability across 17 brain structures (columns). N/D indicates no data was available for those specific regions. Duplicated genes are listed where two data sets were available from the ABA.

The TPR domain-containing family predominately contains proteins that exhibit prolyl-isomerase activity, and these co-chaperones are essential for the ATPase activity of HSPCs. For analysis, we divided this family into known co-chaperones with diverse homologies, FK506 binding proteins, Peptidylprolyl isomerases, and Tetratricopeptide repeat proteins (Figures 4 and 5). Like the DnaJs and their HSPA counterparts, the TPRs have a relatively higher degree of variability than their HSPC counterparts. The TPR genes in the first group that exhibit ubiquitously high expression include Nktr, Puf60, and Hop. Of these three, the most is known about Hop. Hop is a major adaptor protein which coordinates the client protein transfer from HSPA to HSPC and regulates the ATPase activity of each in the transfer process [44]. ‘Hop’ is generally used to distinguish the mammalian version of the gene from the yeast version, stress-inducible protein 1 (STI1), whereas stress-inducible phosphoprotein 1 (Stip1) is typically the term used in proteomics literature. Hop, STI1, and Stip1 are the same gene.

Genes with high levels of variation in expression include Nudc, Ptges3, Ptplad1, and Tomm70a. Ptges3 is also thought to play a major role in protein folding by locking HSPC in an ATP-dependent state which has high affinity for client proteins [45]. Ptplad1, which has a similar expression profile to Ptges3, has been shown to recruit Hsp90 and Fkbp8 into protein folding complexes [46]. Tomm70a is a component of the mitochondrial translocation complex [47]. Note that data for Tomm70a is another example in which different probes for the same gene produce discrepant data.

Within the FK506 binding protein family, approximately half are expressed below the 41^st^ percentile. Fkbp6 and Fkbp15 are the only genes highly expressed across all brain regions. Fkbp6 forms a complex with Gapdh and Hsp90, inhibiting and down regulating Gapdh, but the function of Hsp90 in this complex has not been elucidated [48]. Genes with variable expression levels are Fkbp1b, Fkbp5, and Fkbp16. Fkbp5 modulates the translocation of glucocorticoid receptors to the nucleus upon hormone binding [49]; and one might expect considerable variation in the responses of different neural cell populations to glucocorticoids.

The Peptidylprolyl isomerase (Ppi) proteins contain a cyclophilin motif, named as such due to its inhibition by the immunosuppressant cyclosporin; this small molecule-protein complex then inhibits the phosphatase calcineurin which is how cyclophilin is thought to be an immunosuppresant. This Ppi family exhibits generally low levels of expression with exceptions being Pdia1, Pdia3, Pdia6, Ppia, Ppib, and Ppic. Pdia1, Pdia3, and Pdia6 are localized to the ER. Roles for Ppi genes can be quite diverse, but Ppia and Ppib specifically have roles in HIV-host DNA integration and replication, and activation of peripheral T-lymphocytes, respectively [50], [51].

The Tetratricopeptide repeat domain is typically a 34 residue sequence, which is conserved from bacteria to humans. Proteins with this domain, like the Ppi family, have roles primarily in protein-protein interactions in diverse cellular functions. This family also has generally low levels of expression across all brain regions, but shows more variation than Ppi proteins. Genes with variable expression are Ttc10, 11, 14, 19, and 35. Ttc10 is a centrosomal protein, shown to be important for ciliogenesis and for G1-S transition in non-ciliated cells [52]. Ttc11 has been shown to play a role in mitochondria fission, but independently activates apoptosis through the ER [53]. Functions for Ttc14, 19, and 35 have not yet been elucidated.

### HSPB (small HSPs)

**Figure 6:**
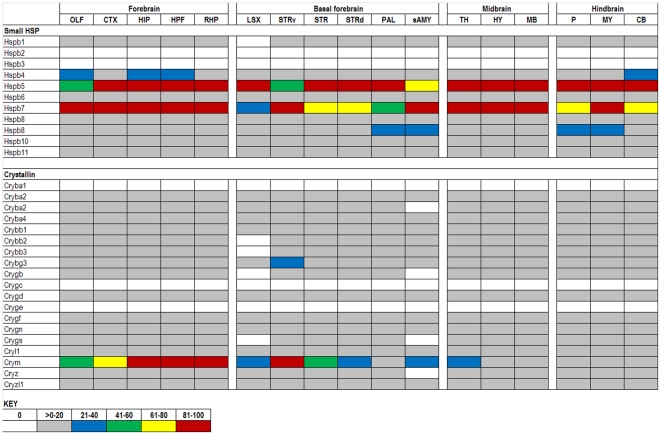
Expression analysis of HSPB family members. The HSPB and Crystallin genes (listed in rows) generally show low levels of expression across structures of the adult mouse brain (columns). Duplicated genes are listed where two data sets were available from the ABA (Hspb8).

Members of the HSPB family, including the crystallins, are generally expressed at low levels (Figure 6). However, Hspb5 (αB-crystallin) and Hspb7 show high levels of basal expression while µ-crystallin (crym) shows variable expression levels. Hspb7 is reported to be induced in aging skeletal muscle [54] while Hspb5 and µ-crystallin are predominately expressed in the mammalian lens and retina, respectively, but also in other non-CNS tissues [55], [56]. Mice deficient in Hspb5 (αB-crystallin) develop normally and are viable [57]; however, these mice develop a skeleton muscle myopathy late in life. Whether the myopathy is due to the absence of Hspb5 is uncertain because the targeted deletion of Hspb5 also inactivates an overlapping gene, Hspb2 that is highly expressed in muscle. The nervous system of these animals appears to be normal [57]. Hspb5 has been shown to inhibit the aggregation of mutant forms of superoxide dismutase 1 linked to familial amyotrophic lateral sclerosis [58], [59]. However, mice that are deficient in Hspb5 while co-expressing mutant SOD1 show only modest changes in disease onset with no obvious change in disease course [59]. Point mutations in other HSPBs, Hspb1 (Hspb2 in mice, Hsp27) and Hspb8 (Hsp22) cause peripheral neuropathies (reviewed in [60]). Dierick and colleagues discuss HSPB-mediated mechanisms other than faulty protein folding which may contribute to neuropathies, including misregulation of apoptosis and collapse of neurofilament network.

### HSPH (HSP100s) and AAA+ATPases

**Figure 7:**
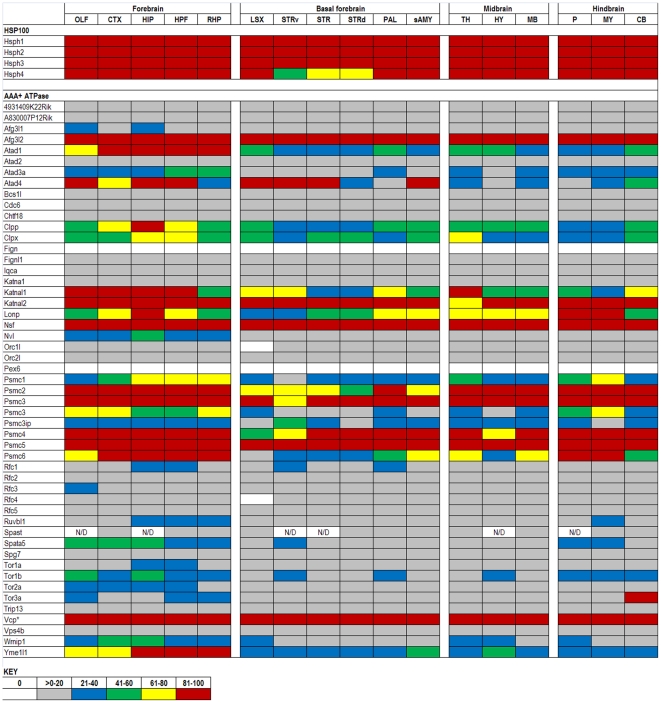
Expression analysis of HSPH family members. HSPH genes (rows) are ubiquitously and highly expressed across all 17 structures of the adult mouse brain (columns). The AAA+ ATPase family members exhibit a more variable pattern. N/D indicates no data was available for those specific regions and, duplicated genes are listed where two data sets were available from the ABA.

Another group of genes that encode proteins involved in the folding of client proteins are high molecular weight HSPHs and the AAA+ ATPases (Figure 7). The HSP100s have high homology to the HSPAs and have been shown to serve as their nucleotide exchange factors [61], [62]. This essential activity for HSPAs might explain why all four members of the HSPHs are highly expressed across the brain.

Whether all of the AAA+ ATPase genes encode proteins that exhibit chaperone-like activity is unknown, but there are clear examples that some of these gene products are involved in the folding of specific substrates [10]. Although almost all of the AAA+ ATPase genes that were examined are expressed at some level, approximately half of the genes are expressed at or below the 20^th^ percentile with only six AAA+ ATPase genes showing expression at high levels across the major brain regions (Figure 7). Among the seven AAA-ATPase genes showing the greatest degree of variability are Atad1, Atad4, Psmc6, and Yme1l1. Very little is known about the Atad family, as they are named only by containing the conserved AAA+ domain. Yme1l1, a homolog of the bacterial gene Ftsh, is better known as an ATP-dependent metalloprotease. Its proteolytic activity in bacteria is documented to be involved in regulating the sigma(32) subunit of RNA polymerase, which, under stressed conditions, is mediated through interactions with DnaK and DnaJ (Hsp70 and Hsp40 homologues, respectively) [63]. Psmc6 is one of six ATPase subunits of the 19S subcomplex – the regulatory subunit of the 26S proteasome. Given that Psmc6 serves such a housekeeping function, it is surprising to observe variation in expression levels. Another interesting gene is Tor3a which is highly expressed in the CB, but expressed at low levels (<21) in almost every other region. Tor3a is a member of the Torsin family. Mutations in the Tor1a gene (an inframe deletion of a single glutamic acid) cause early onset dystonia [64], [65]. Although the function of Tor3a is unclear, it is known to be upregulated in response to interferons [66].

### Chaperonins (HSP60s)

**Figure 8:**
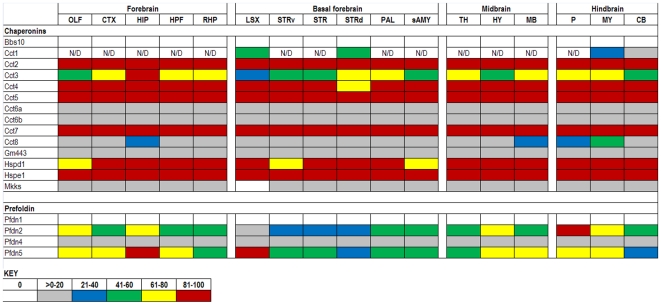
Expression analysis of Chaperonin family members. Expression levels of the chaperonin and HSPD/E genes. The two major families of the chaperonin genes are the Chaperonins and Prefoldins (rows). With a few exceptions, they exhibit a bimodal expression pattern across 17 structures of the adult mouse brain (columns). N/D indicates no data was available for those specific regions.

There are two chaperonin subfamilies: 1) the traditional GroEL/Hsp60/Hspd1 and GroES/Hsp10/Hspe1 partners, and 2) the Cytosolic chaperonin containing t-complex (Cct/TRiC) which function by encapsulating proteins and domains of proteins up to 220 kD in size to facilitate folding. In general, these genes are uniformly expressed across brain regions (Figure 8) and are either abundantly expressed (6 of 14 in the top 20^th^ percentile) or poorly expressed (6 of 14 in the bottom 20^th^ percentile); Cct subunits are not stress inducible. Among the more highly expressed are Hspd1, Hspe1, and Cct subunits 2, 4, 5, and 7. Hspd1 and Hspe1 function to refold proteins once they have been translocated to the mitochondrial matrix. Mutations in Hspd1 has been linked to hereditary spastic paraplegia 13 [67] and a hypomyelination disorder [68].

Within the Cct subfamily, it is surprising that subunits of a heteromeric octamer are not uniformly expressed at identical levels (Figure 8). Cct substrates are primarily housekeeping cytoskeletal proteins such as actin, tubulin, and β-propeller containing proteins; however non-cytoskeletal proteins are also known to be substrates [11], [69]. Not much is known about their individual function. A point mutation in Cct 4 leads to hereditary sensory neuropathy in rats [70] and a point mutation in Cct 5 also leads to a sensory neuropathy in humans [71]. It has been suggested that a general inability to properly fold cytoskeletal elements in the periphery leads to neuropathies.

The Cct subfamily is aided by co-chaperones, the prefoldins. Prefoldins (Pfdn/GimC) function by binding newly synthesized, partially folded proteins from the ribosome and guiding them exclusively to the Cct complex [72], [73]. Examples of variable expression levels include Prefoldins 2 and 5, but very little is known about their individual function. Humans express two additional prefoldins, Pfdn3 and Pfdn6, which are absent in mice.

### HSF

**Figure 9:**
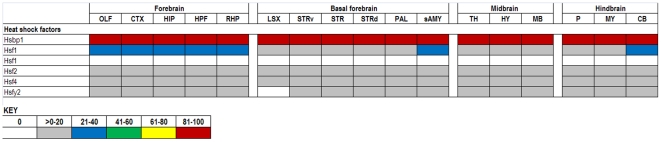
Expression analysis of HSF family members. HSF proteins generally show low levels of expression whereas HSF binding protein is highly expressed. Duplicated genes are listed where two data sets were available from the ABA (Hsf1).

The major regulators of the inducible chaperones are the Heat Shock Factors (HSFs). All of the HSFs expressed in brain are expressed at low levels (Figure 9). Interestingly, a negative regulator of HSFs, HSF-binding protein 1 (Hsbp1) [74], is highly expressed in all brain regions. Hsf1 is rapidly responsive to extra- or intracellular signals to activate transcription, but the response can be halted just as quickly by Hsbp1 disrupting the trimerization of Hsf1 thus preventing its binding to heat shock elements (HSEs) [75]. Hsf1 activity is also regulated by the deacetylase SIRT1; SIRT1 removes the acetyl group from Hsf1 K80 permitting binding to the Hsp70 promoter [76]. Knockout mice for Hsf1 are not able to induce transcription of Hsp25/27, Hsp70, or Hsp60, are of reduced size, and females are infertile [77]. Hsf2 is known to be developmentally regulated, expressed broadly until 15.5 days of gestation, but is restricted to the CNS the rest of development. Hsf2 knockout mice have some embryonic lethality, reduced fertility, and exhibit some CNS defects [78], [79]. However, these phenotypes have not been reproduced by others [80]. While Hsf2 was shown to bind HSEs *in vivo*, there was no correlation to the expression of other HSPs [81]. However, it has been suggested by *in vitro* studies that Hsf1 and Hsf2 coordinate the response to heat shock [82]. Hsf4 is thought to be a negative regulator of Hsf1-mediated transcription [83]. Mice nullizygous for Hsf4 develop cataract with abnormal lens fiber cells due to the overexpression of gamma-crystallin genes [84]. These authors also found Hsf1 and Hsf4 compete in regulating fibroblast growth factor expression in tissue beyond the lens, regulating cell growth and differentiation. While the general heat shock response is mostly uniform across cell types, there is a trend for neurons to be more susceptible to these stressors [15], [85].

### Others

**Figure 10:**
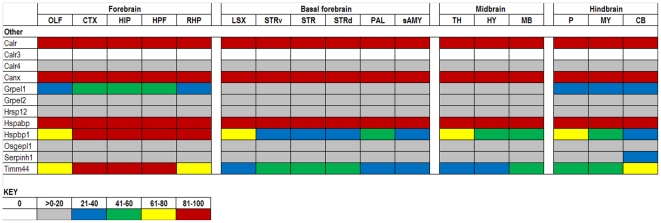
Expression analysis of other uncategorized chaperones. Expression levels are shown of genes that function within the chaperone network but do not succinctly fit in the aforementioned families are categorized as ‘Other.’

A number of proteins, thought to function in a chaperone pathway, do not fit precisely into previous categories and are discussed in this final group (Figure 10). Hspbp1 is one of a small group of nucleotide exchange factors for HSPA proteins that can also confer client protein specificity (reviewed in [37]). Hspbp1 is not inducible; it is highly expressed in the forebrain with lower levels in the cerebellum and striatum. Another gene with a similar expression pattern is Timm44, which receives proteins from Hspa8 to translocate into the mitochondria. Hspabp, thought to be a tumor suppressor, is important for Hsp70 function by mediating its interaction with Hsp90 [86]. Hspabp and three members of HSPC are highly expressed throughout the brain but no reports were found of coordinated regulation. Overexpression of Hspabp was shown to decrease inclusion formation in an cell culture model of spinal and bulbar muscular atrophy [87]. With additional experiments, these authors concluded this decrease was due to interactions with constitutive Hsc70 and the prevention of protein misfolding.

### Expression by brain region

To draw further analyses regarding brain regions and levels of HSP expression, we grouped the 17 structures provided by the ABA into four major groups: forebrain, basal forebrain, midbrain, and hindbrain. We found 62 genes that exhibited high levels of expression in all five substructures of the forebrain that were analyzed (Table 3 and Figure S1). However, we found only 14 genes in which expression in the forebrain was significantly higher, or enriched, relative to all other brain regions. The specific number of genes is different for each of the four major brain groups. In the basal forebrain, midbrain, and hindbrain, we identified 38, 51, and 53 genes that were highly expressed, respectively (Figures S2, S3, S4). However, the number of genes in which there was enriched expression in these regions, relative to all other regions, was 0, 0, and 2, respectively. This finding suggests that most of the chaperones that are highly expressed in one specific region of the brain are usually expressed at ubiquitously high levels throughout the brain.

**Table 3:** Summary of Chaperone Gene Expression by Brain Region.

Brain region	HSPA	DnaJ	HSPC	TPR	HSPB	HSPH	Chaperonins	HSF	Other	Total
High forebrain	4	12	3	21	1	12	5	1	3	62
Enriched forebrain	1	4	0	5	0	3	0	0	1	14
High basal forebrain	3	6	3	10	0	8	4	1	3	38
Enriched basal forebrain	0	0	0	0	0	0	0	0	0	0
High midbrain	3	7	3	16	2	10	6	1	3	51
Enriched midbrain	0	0	0	0	0	0	0	0	0	0
High hindbrain	4	8	3	15	1	12	6	1	3	53
Enriched hindbrain	0	1	0	0	0	1	0	0	0	2

This table summarizes expression levels across different brain regions. The table indicates the number of genes that are highly expressed in major subdivisions of the brain and the number that are specifically highly expressed in a given brain region. Fourteen chaperone genes are highly enriched in the forebrain. Two chaperones are enriched in hindbain.

### Subcellular distributions of chaperones

Heat shock proteins across all families are known to localize to specific regions within the cell. For reviews regarding chaperone activity in the cytosol, mitochondria, and ER, the reader is directed to Young *et al*., 2004, Voos *et al*., 2002, and Qiu *et al*., 2006 [7], [88], [89]. Within the brain, most of the expressed genes of the chaperone network are cytosolic proteins (Table 4 and Figure S5). One well-studied functional cluster of genes involved in the folding of cytosolic proteins includes Hspa8, a variety of DnaJs, nucleotide exchange factors Bag1 and Hspbp1 (releasing Hspa8 of ADP), and Hspabp, a stabilizing factor for ADP-bound Hspa8. Hspa8 and Hspabp are both ubiquitously expressed at high levels, suggesting Hspa8 is usually stably holding client proteins in an ADP-bound state. To rapidly release client proteins, ADP is released from Hspa8 by Hspbp1 or Bag1. Hspbp1 is only highly expressed in the forebrain and Bag1 is expressed at low levels across all brain regions, suggesting that the forebrain may be more active in cytosolic chaperone activity. Additionally, the DnaJs, which activate Hspa ATP hydrolysis, are only moderately expressed in the forebrain and exhibit lower levels of expression in the other regions. By contrast, the functional cluster of chaperones in the ER is relatively uniformly expressed across different regions of the brain (either ubiquitously high or ubiquitously low).

**Table 4:** Summary of Chaperone Subcellular Localization.

Subcellular location	HSPA	DnaJ	HSPC	TPR	HSPB	HSPH	Chaperonins	HSF	Other	Total
Cytosol	2	6	1	11	3	3	9	0	2	37
Endoplasmic reticulum	1	7	1	6	0	1	0	0	3	19
Mitochondria	1	4	0	1	0	2	2	0	2	12
Endosomes	1	3	0	0	0	0	0	0	0	4
Nuclei	0	1	0	0	3	0	0	3	0	7
Other*	1	0	0	0	2	0	0	0	0	3

The number of genes within each of the nine families that localize to a specific subcellular locations are shown.

*The category ‘Other’ combines a number of genes found to localize to the plasma membrane or microsomes. Most chaperones are localized to the cytosol.

### Validation of ABA expression data

Within the existing literature, there are a number of studies that provide corroborating data to validate the expression data in the ABA. First, the ABA expression data demonstrates little or no expression for chaperones that are known to be inducible. For example, the inducible Hsp70 (Hspa1a) is present at extremely low levels in brain, whereas the constitutively expressed Hsp90, Hsc70 an Hsp60 are easily detected [90]. Data that corroborate the inducible and constitutive expression of Hspa1a (Hsp70) and Hspa8 (Hsc70), respectively, has been shown by western blot of rat brain regions [91]. Control or heat-shocked rat brains were dissected into cerebral cortex, hippocampus, midbrain, and spinal cord. Homogenates of each structure were analyzed by SDS-PAGE and western blots for various chaperones. Hspa8 is similarly expressed in all regions and both conditions while Hspa1a is expressed in all regions only after heat shock. The ABA scores Hspb2/Hsp27 as not expressed in any brain region (Figure 6). Immunoblot studies have demonstrated that baseline expression of Hspb2 is almost undetectable in the rat cortex, hippocampus, and midbrain [91]. They also showed that Hspb2 is inducible, however, to detectable levels in brain. Other studies have used RT-PCR, *in situ* hybridization, and immunoblots across various mouse organs [92]. In the brain, Hspb1, 5, 6, and 8 were found at the mRNA and protein level. The ABA scores all four of these genes as expressed at some level although Hsbp5 is scored as being more abundant than the other three genes (Figure 6). Hspb5 was also seen to be higher by RT-PCR analysis but not by protein [92]. In studies of Hspb7 expression in mouse brain, RT-PCR data from Quraishe and colleagues is consistent with the ABA in that Hspb7 mRNA is detected [92]. Note that the ABA scores expression of Hspb7 as being very high in forebrain (Figure 6). However, Quraishe reported that the protein could not be detected. Assuming that the antibody used was sensitive enough to detect the levels of protein present, this study illustrates the need to verify mRNA expression data with protein expression data. Together, these reports indicate that the ABA is consistent with the literature with regards to mRNA; however, there are instances where the ABA does not predict protein levels.

**Figure 11:**
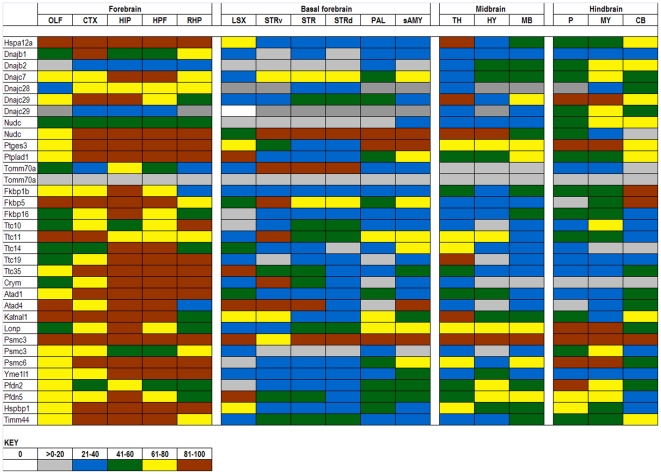
Summary of chaperone genes showing variable levels of expression. The 30 genes that are variably expressed across ≥2 brain regions are shown. Genes from all groups except HSPC and HSF are represented. Duplicated genes shown are instances where the ABA had two data sets. Generally, variably expressed genes are higher in the forebrain and lower in the rest of the brain.

**Figure 12:**
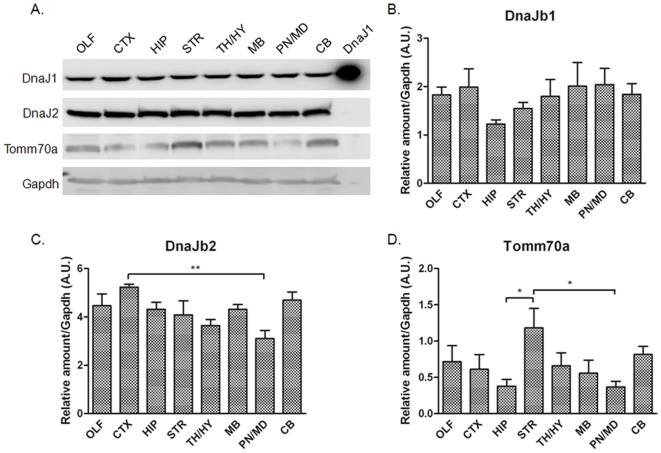
Immunoblot analysis of DnaJb1, DnaJb2, and Tomm70a. A. Immunoblots of different brain regions. Top Panel – A positive control sample for the antibody was loaded in the last late (labeled DnaJ1). Quantitation for DnaJb1, DnaJb2, and Tomm70a are shown in panels B–D, respectively.

From our analysis of the chaperone expression data in ABA we can identify about 30 genes in the ABA that show a significant degree of variability in levels across different structures of the brain (Table 1, Figure 11, Table S3). Clearly it would be worthwhile to know whether the variability in mRNA levels across different brain structures translates into differing levels of protein. Most of the genes we identify as variable are poorly characterized in terms of function or protein levels. For many we could not identify validated antibody reagents that are required for accurate determination of protein levels. However, we identified antibodies for 3 genes that show a broad range of expression levels, from the lowest quintile to the highest quintile: DnaJb1, DnaJb2, and Tomm70a (Figure 11). Immunoblots of homogenates from different brain structures revealed some discrepancies between the ABA predictions based on mRNA levels and protein levels (Figure 12A). In contrast to predictions based on the ABA database, protein levels of DnaJb1 were not variable across brain structures (Figure 12B). For DnaJ2b we detected a statistically significant difference in protein levels between the cortex and the pons/medulla (Figure 12C), but ABA predicts these structures should have similar levels of expression (Figure 11). For Tomm70a the ABA contains expression data for two different probe sets, one of which predicts uniformly low levels of expression and one that predicts varied levels of expression (see Figure 4). Immunoblots for Tomm70a showed varied levels of expression with the variability closely matching what is predicted by the ABA (Figure 12D). The levels ofTomm70a were highest in striatum with statistically significant lower levels in hippocampus and pons/medulla. Thus, for 2 out of 3 of the chaperones in which we examined protein levels, we find differences from what is predicted by the ABA. It is possible that the inconsistencies between the levels of mRNA for DnaJb1 and DnaJb2 protein may be explained by subcellular localization of the proteins. The mRNA for these proteins is predominately localized to neuronal cell bodies, but these soluble cytosolic proteins are likely to be transported down axons possibly distributing the protein across many structures of the brain. In the case of Tomm70a, the protein is localized to the outer membrane of mitochondria [47], which are very abundant in cell bodies. From these data, it is hard to judge the extent to which the expression data in the ABA predicts protein levels in brain. It is possible that there is even less variability in the levels of chaperones across brain structures than is predicted by the ABA.

### Conclusions

An analysis of expression data from the Allen Brain Atlas provides insight into the steady state regulation of gene expression from the chaperone network in the murine brain. About one-third of the genes of the chaperone network are expressed at low levels or not at all across the entire brain. We can be fairly confident that an absence or low levels of mRNA should be a good predictor for low levels of protein expression. However, some of these genes that are expressed at low levels in normal brain may be induced by various stimuli or disease states. The ABA predicts uniformly high levels of expression for 35 genes of the chaperone network. Whether the levels of protein for these 35 genes are uniformly high is uncertain because a myriad of post-transcriptional regulatory mechanisms could suppress translation of the mRNA or diminish the abundance of protein. However, among the 35 genes that are uniformly highly expressed are several essential house-keeping genes such as Hsc70, BiP, Hsp90, calreticulin, and calnexin. For the genes at these extremes, it is likely that the ABA provides fairly accurate predictions of protein levels.

Between these extremes are a number of genes that show variability across the structures of the brain. At the level of transcriptional regulation, we observe the greatest diversity in expression of DnaJ, TPR co-chaperones, and AAA+ATPases. This finding is not too surprising as it is thought that the products of these genes are primarily responsible for bringing protein clients to chaperone activities [93]; and one might expect significant diversity in regulating the folding of both unique and ubiquitous protein clients across the many diverse cell populations of the brain. However, although these expression data indicate diversity, the differences in expression levels that encode the unique signature of a neuronal population are relatively subtle. For example, between the cerebellum and cortex only 75 of the 270 genes show differences of any magnitude and of these only 6 differ in expression by values that would seem to be significant (greater than 2 quintiles in level). Of course, it is possible that post-transcriptional or post-translational regulatory processes could increase the variation in functional levels of these factors (both negatively and positively) in different neuronal populations. It is also possible that other systems involved in maintaining protein homeostasis modulate the network by regulating synthesis and degradation of some of these chaperones. Moreover, all of the chaperone network systems function as multi-protein complexes. Thus assembly of functional chaperones (particularly for the DnaJ and TPR co-chaperones) provides an opportunity to increase diversity in function. However, for each of these chaperone/co-chaperone systems, we observe relatively little variation in the expression levels of the chaperone component. For example, the DnaJ proteins that function as co-chaperones in selection of client proteins for the HSPA chaperones (HSP70s), the expression levels of the HSPA chaperones varies little (see Figure 1). A similar parallel applies to the TPR co-chaperone complexes. Thus although the formation of multiprotein complexes could produce greater diversity in function, many of the essential components of the complex are relatively uniformly expressed.

In summary, our meta-analysis organizes expression levels of mRNA for components of the chaperone network in various regions of the brain. Somewhat surprisingly, nearly a third of genes in the chaperone network are expressed at very low levels or not at all. Of the genes that are expressed, relatively few show much variation in mRNA levels across structures of the brain. Although there could be post-transcriptional mechanisms that increase variability in levels of protein for these genes, the levels of mRNA provide a view of the landscape of their regulation. As upregulation of the heat shock response gains momentum as a therapeutic intervention in neurodegenerative disease, it will be important to determine which of the genes detailed here respond to such therapeutics and whether selective modulation of subsets of chaperones might lead to disease specific interventions. Given the plethora of data provided by the ABA, it would make sense to use an *in situ* hybridization identical to the protocols detailed on the website as at least one approach to characterize how a specific compound or insult affects expression of the chaperone network.

## Supporting Information

Figure S1 Chaperones exhibiting high forebrain expression. Top portion: Genes that exhibited highest expression (red) in all five regions of the forebrain, irrespective of expression levels in other regions are compiled. Bottom portion: Genes that exhibited highest expression in 4 out of 5 forebrain regions and had fewer than three other regions with highest expression were considered to be enriched. See Table 3.
(0.24 MB TIF)

Figure S2 Chaperones exhibiting high basal forebrain expression. Top portion: Genes that exhibited highest expression (red) in all six regions of the basal forebrain, irrespective of expression levels in other regions are compiled. Bottom portion: No genes were found that exhibited highest expression in 5 out of 6 basal forebrain regions and had fewer than three other regions with highest expression. See Table 3.
(0.13 MB TIF)

Figure S3 Chaperones exhibiting high midbrain expression. Top portion: Genes that exhibited highest expression (red) in all three regions of the midbrain, irrespective of expression levels in other regions are compiled. Bottom portion: No genes were found that exhibited highest expression in 2 out of 3 midbrain regions and had fewer than four other regions with highest expression. See Table 3.
(0.17 MB TIF)

Figure S4 Chaperones exhibiting high hindbrain expression. Top portion: Genes that exhibited highest expression (red) in all three regions of the hindbrain, irrespective of expression levels in other regions are compiled. Bottom portion: Genes that exhibited highest expression in 2 out of 3 hindbrain regions and had fewer than four other regions with highest expression were considered to be enriched. See Table 3.
(0.18 MB TIF)

Figure S5 Subcellular localization of chaperones. Genes known to localize to specific subcellular compartments are organized into cytosol, endoplasmic reticulum, mitochondria, microsome, endosome, nucleus, and plasma membrane regions. N/D indicated no data was available from the ABA. (*) Serpinh1 has chaperone activity but is a member of the serpin family, of which no other members have documented chaperone activity. See Table 4.
(1.26 MB TIF)

Table S1 Complete list of all chaperones identified. This table lists the full name of each gene classified herein as a chaperone. The table also lists the abbreviations used and an alias or reason the ABA was unable to provide data (ABA status), where applicable. Genes are organized into nine groups and listed alphabetically.
(0.06 MB XLS)

Table S2 List of genes that are uniformly expressed across brain structures. The genes that are ubiquitously expressed at high and low levels are listed as well as genes that fail to show evidence of expression.
(0.07 MB DOC)

Table S3 Hyperlinks to ABA data on 30 differentially expressed chaperones. This table lists the 30 variably expressed genes and the webpage address from the ABA. Each webpage address shows the primary data we used to compile our lists and determine the variability in expression levels.
(0.03 MB XLS)
