# Supplementary figures and images for: Analysis of Chaperone mRNA Expression in the Adult Mouse Brain by Meta Analysis of the Allen Brain Atlas
Source: PLoS One. 2010 Oct 28;5(10):e13675. doi: 10.1371/journal.pone.0013675 (PMC2965669; doi:10.1371/journal.pone.0013675)

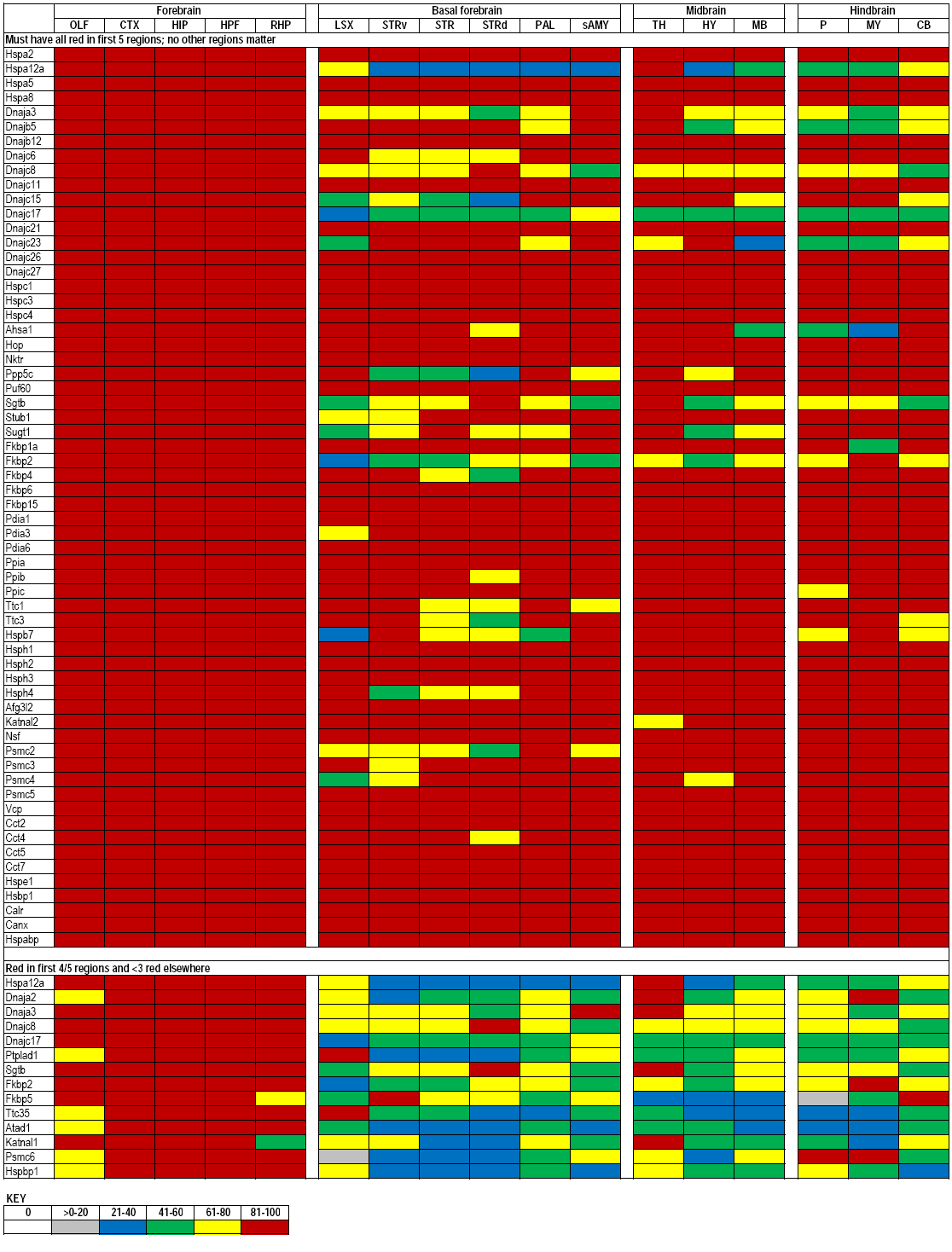

Supplement: Figure S1 — Chaperones exhibiting high forebrain expression. Top portion: Genes that exhibited highest expression (red) in all five regions of the forebrain, irrespective of expression levels in other regions are compiled. Bottom portion: Genes that exhibited highest expression in 4 out of 5 forebrain regions and had fewer than three other regions with highest expression were considered to be enriched. See Table 3. (0.24 MB TIF) [file pone.0013675.s001.tif]

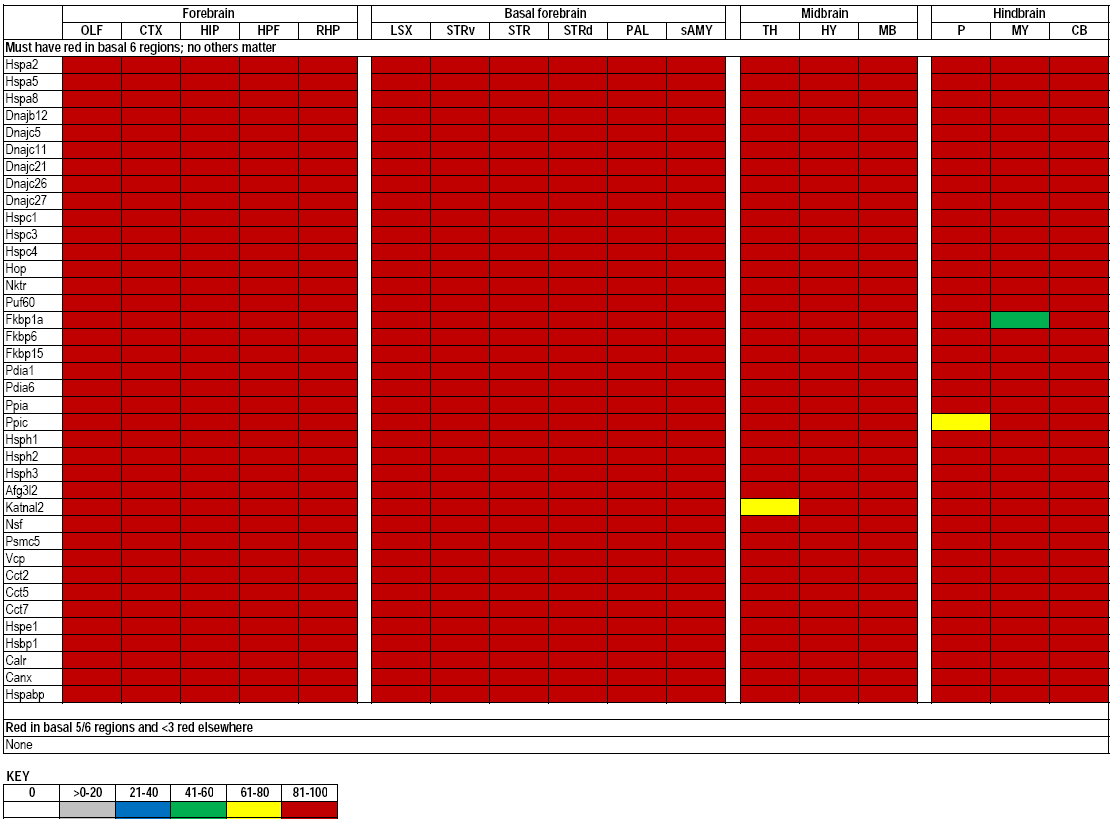

Supplement: Figure S2 — Chaperones exhibiting high basal forebrain expression. Top portion: Genes that exhibited highest expression (red) in all six regions of the basal forebrain, irrespective of expression levels in other regions are compiled. Bottom portion: No genes were found that exhibited highest expression in 5 out of 6 basal forebrain regions and had fewer than three other regions with highest expression. See Table 3. (0.13 MB TIF) [file pone.0013675.s002.tif]

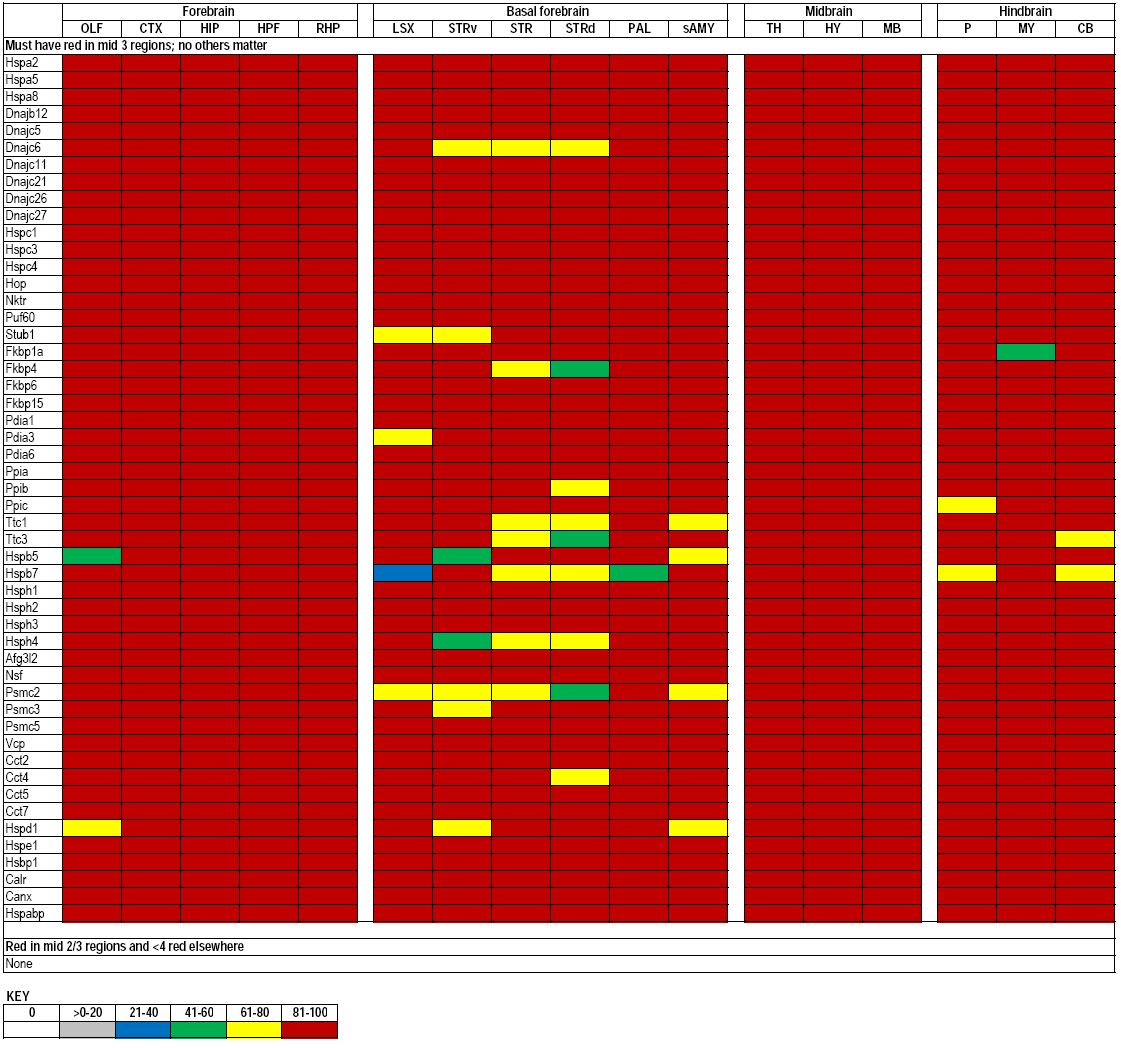

Supplement: Figure S3 — Chaperones exhibiting high midbrain expression. Top portion: Genes that exhibited highest expression (red) in all three regions of the midbrain, irrespective of expression levels in other regions are compiled. Bottom portion: No genes were found that exhibited highest expression in 2 out of 3 midbrain regions and had fewer than four other regions with highest expression. See Table 3. (0.17 MB TIF) [file pone.0013675.s003.tif]

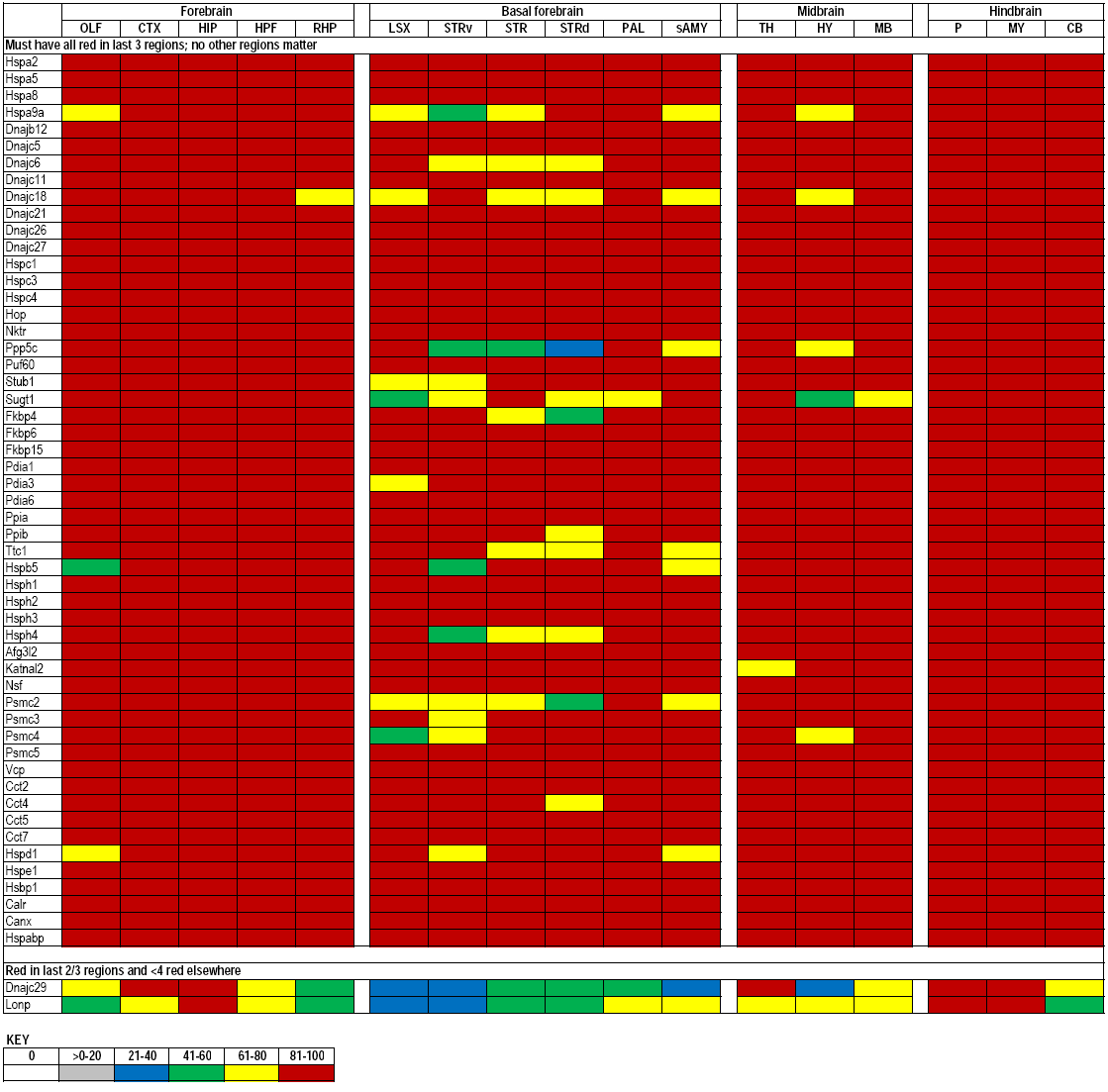

Supplement: Figure S4 — Chaperones exhibiting high hindbrain expression. Top portion: Genes that exhibited highest expression (red) in all three regions of the hindbrain, irrespective of expression levels in other regions are compiled. Bottom portion: Genes that exhibited highest expression in 2 out of 3 hindbrain regions and had fewer than four other regions with highest expression were considered to be enriched. See Table 3. (0.18 MB TIF) [file pone.0013675.s004.tif]

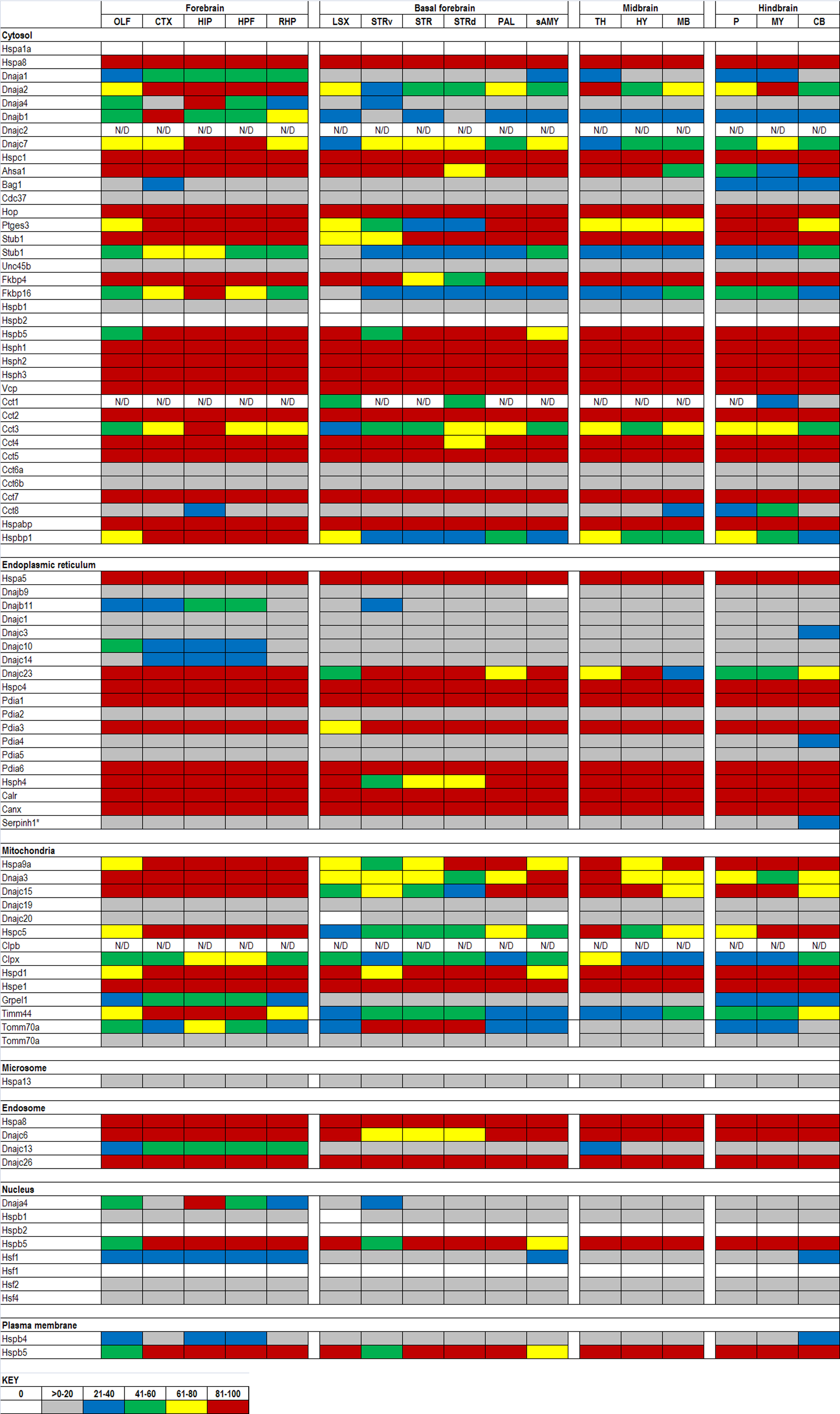

Supplement: Figure S5 — Subcellular localization of chaperones. Genes known to localize to specific subcellular compartments are organized into cytosol, endoplasmic reticulum, mitochondria, microsome, endosome, nucleus, and plasma membrane regions. N/D indicated no data was available from the ABA. (*) Serpinh1 has chaperone activity but is a member of the serpin family, of which no other members have documented chaperone activity. See Table 4. (1.26 MB TIF) [file pone.0013675.s005.tif]
