# Supplementary material for: Analysis of Chaperone mRNA Expression in the Adult Mouse Brain by Meta Analysis of the Allen Brain Atlas
Source: PLoS One. 2010 Oct 28;5(10):e13675. doi: 10.1371/journal.pone.0013675 (PMC2965669; doi:10.1371/journal.pone.0013675)
Supplement: Table S2 — List of genes that are uniformly expressed across brain structures. The genes that are ubiquitously expressed at high and low levels are listed as well as genes that fail to show evidence of expression. (0.07 MB DOC) [file pone.0013675.s007.doc]

Table S2. List of genes showing uniform expression across brain structures.

| **Ubiquitously High** | **Ubiquitously Low (1-20 quintile)** | **Ubiquitously Off (not detected)** |
| --- | --- | --- |
|  |  |  |
| **HSP Family** | **HSP Family** | **HSP Family** |
| Hspa2 – Hsp70 family | Hspa 13 – Hsp70 family | Hspa1a – Hsp70 inducible |
| Hspa5 – Grp78,BiP | Dnajb7, 8, 13 - Hsp40 family B | Cryba1 – Crystallin, beta 1 |
| Hspa8 - Hsc70 | Dnajc1, 5b, 5g, 16, 19, 22, 25 - Hsp40 family C | Crygc, e – Crystallin, gamma C, E |
| Dnajb12 – Hsp40 family B | Fls485 – Hsp40 family | Hspb2, 3 – sHSP family |
| Dnajc5, 11, 21, 26, 27 - Hsp40 family C | Cryba2, 4 – Crystallin, beta A |  |
| Hspc1 – Hsp90 alpha | Crybb1 – Crystallin, beta B |  |
| Hspc4 – Hsp90b1 (Grp94) | Crygd, f, n – Crystallin, gamma |  |
|  | Cryl1 – Crystallin, lambda |  |
|  | Cryzl1 – Crystallin, zeta like 1 |  |
|  | Hspb8 – sHsp family |  |
|  |  |  |
| **TPR family** | **TPR family** | **TPR family** |
| Hop – Hsp70/Hsp90 BP | Cdc37 | Fkbp11 – FK506 binding protein |
| Nktr - | Fkbp7, 10 – FK506 binding protein | PpiL3 – Prolyl isomerase like |
| Puf60 – splicing factor | Fkbpl – FK506 binding protein | Ttc37 – tetratricopeptide repeat |
| Fkbp15 - FK506 BP | Itgb 1bp2 – Integrin β1-binding protein 2 |  |
| Pdia1, 6 – disulfide isomerase | Nasp – Nuclear Autoantigenic sperm protein |  |
| Ppia – prolyl isomerase A | Pdia2, 5 - disulfide isomerase |  |
|  | Pih 1d2 |  |
|  | Ppig, h - prolyl isomerase G, H |  |
|  | Ppil1, 4, 5 - prolyl isomerase like-1, 4, 5 |  |
|  | S100a1 – S100 Ca BP A1 |  |
|  | Tomm70a – Mitochondrial membrane translocase * |  |
|  | Ttc7, 8, 9, 9c, 13, 16, 18, 23, 24, 25, 28, 29, 34, 36,38 – Tetratricopeptide repeat domain proteins |  |
|  | Unc45b |  |
|  |  |  |
| **Ubiquitously High** | **Ubiquitously Low (1-20 quintile)** | **Ubiquitously Off (not detected)** |
|  |  |  |
| **AAA-ATPase** | **AAA-ATPase** | **AAA-ATPase** |
| Afg312 | Atad2 - | Fign – Fidgetin |
| Hsph1 – Hsp105 | Bcs1L | Pex6 – Peroxisomal biogenesis factor |
| Hsph2 – Hsp4 | Cdc6 |  |
| Hsph3 – Hsp4-like | Chtf18 |  |
| Nsf | FignL1 |  |
| Psmc5 | Iqca |  |
| Vcp | Orc21 |  |
|  | Rfc2, 5 – Replication factor C |  |
|  | Spg7 – Spastic paraplegia 7 homolog |  |
|  | Trip13 - |  |
|  | Vps4b |  |
|  | 4931409K22Rik |  |
|  | A830007P12Rik |  |
|  |  |  |
| **Chaperonins** | **Chaperonins** | **Chaperonins** |
| Cct2, 5, 7 – chaperonin subunits | Cct6a, 6b – chaperonin subunits | Bbs10 – Bardet-Biedl syndrome 10 |
| Hspe1 – Hsp 1 (chaperonin 10) | Gm443 | Pfdn1 – Prefoldin 1 |
|  | Prdn4 – prefoldin |  |
|  |  |  |
| **Heat Shock Factors** | **Heat Shock Factors** | **Heat Shock Factors** |
| Hsbp1 Hsf BP1 | Hsf2, 4 | Hsf1 |
|  |  |  |
| **Other** | **Other** | **Other** |
| Calr - Calreticulin | Calr4 – Calreticulin | Calr3 - Calreticulin |
| Canx - Calnexin | Grpe12 – Glucose regulated protein E-like |  |
| Hspabp – Hsp70 BP | Hrsp12 - Heat responsive protein 12 |  |
|  | OsgepL1 – O-sialoglycoprotein endopeptidase-like 1 |  |
